# Patterns of Gene Expression in Cutaneous T-Cell Lymphoma: Systematic Review of Transcriptomic Studies in Mycosis Fungoides

**DOI:** 10.3390/cells10061409

**Published:** 2021-06-06

**Authors:** Melika Motamedi, Maggie Z. X. Xiao, Aishwarya Iyer, Robert Gniadecki

**Affiliations:** 1 Division of Dermatology, Department of Medicine, University of Alberta, Edmonton, AB T6G 2R3, Canada; melika@ualberta.ca (M.M.); zixuan2@ualberta.ca (M.Z.X.X.); aiyer2@ualberta.ca (A.I.); 2 8-112 Clinical Sciences Building, University of Alberta, Edmonton, AB T6G 2G3, Canada

**Keywords:** cutaneous T-cell lymphoma (CTCL), mycosis fungoides (MF), transcriptome

## Abstract

Mycosis fungoides (MF) is the most prevalent type of skin lymphoma. In its early stages, it has a favorable prognosis. However, in its late stages, it is associated with an increased risk of mortality. This systematic review aimed to identify the transcriptomic changes involved in MF pathogenesis and progression. A literature search was conducted using the database PubMed, followed by the extraction of 2245 genes which were further filtered to 150 recurrent genes that appeared in two or more publications. Categorization of these genes identified activated pathways involved in pathways such as cell cycle and proliferation, chromosomal instability, and DNA repair. We identified 15 genes implicated in MF progression, which were involved in cell proliferation, immune checkpoints, resistance to apoptosis, and immune response. In highlighting the discrepancies in the way MF transcriptomic data is obtained, further research can focus on not only unifying their approach but also focus on the 150 pertinent genes identified in this review.

## 1. Introduction

Mycosis fungoides (MF) is the most common cutaneous T-cell lymphoma (CTCL) derived from CD4+ T-cells [1]. MF initially presents as erythematous patches and plaques and, in those early stages (IA-IB), the disease has a favorable prognosis. However, approximately 25% of patients progress to the advanced stages, characterized by cutaneous tumors or erythroderma, lymph node and blood involvement, occasionally organ involvement, and a dramatic reduction in five-year survival from approximately 80% to 25% [2,3,4].

Omics approaches are widely used to elucidate the mechanisms of cancerogenesis and identify druggable targets for personalized treatment of cancer. Large open-access databases, such as The Cancer Genome Atlas (TCGA), allow exploration of the genome-wide expression of individual genes in different tissues and cancers from thousands of patients and aids in identifying several prognostic biomarkers [5,6]. However, those resources focus on common cancers and have been less helpful for rare malignancies, such as MF. In particular, the molecular mechanisms responsible for the stage progression of MF have turned out to be challenging to define because of the vast genetic and transcriptomic heterogeneity of the disease. Despite thousands of non-synonymous mutations described in CTCL, very few are recurrent and even fewer could be classified as the true drivers of the disease [7,8,9,10,11].

Leaving aside the low number of MF patients reported to the open-access databases, there are several barriers to the efficient analysis of the transcriptomic data. First, the clinical information is often insufficient. Some studies lump the data from different CTCLs and do not provide the clinical diagnosis, neglecting the fact that different CTCLs differ in their pathogenesis, clinical manifestations, and prognosis. Historically, Sézary syndrome (SS) was understood to be the more advanced form of MF; however, it is now recognized that these two diseases arise from two distinct cell types; SS from central memory T-cells and MF from effector memory T-cells [12]. This biological distinction explains the differing molecular profile of these two diseases [13] and emphasizes the need to examine MF and SS in their classic forms as separate entities, rather than clustering them onto a disease continuum of moderate to severe stage disease, respectively. Furthermore, the TNMB stage is rarely reported and, instead, the authors used arbitrary designations such as “early-stage” or “late-stage” without providing clear definitions. Some papers refer to stage IA to IB (both patch/plaque lesions with no lymph node involvement) as early-stage [14,15], whereas others include stage IIA (involved abnormal peripheral lymph nodes) as early-stage [16,17]. Other studies combine all stages from plaque, through tumors, to generalized erythema in their analysis [18,19] 

Second, crude skin biopsies are commonly used to study transcriptomics, without taking into account that malignant lymphocytes may comprise only a tiny fraction (<10%) of the cells in the skin sample [7]. Thus, most detected transcripts are derived from non-lymphoid cells, or the inflammatory infiltrate accompanying cancer cells, and it is almost impossible to catalog the tumor-specific transcripts. Third, there is no consensus regarding the control samples to which the differentially expressed genes are compared. Often, normal skin from healthy individuals serves as control [14,19,20,21], but this can be criticized because the bulk of the signal in normal skin comes from non-lymphoid cells such as keratinocytes or mesenchymal cells. Perhaps a better control is biopsies from inflammatory skin conditions (e.g., psoriasis or contact dermatitis) [17,22], however, it is unclear which type of inflammation would be the best reference. Isolated CD4+ T-cells are sometimes used as cell-specific control [23], but it is unclear whether quiescent or activated T-cells would be preferred. Lastly, the diversity of methods used in different studies further complicates the ability to compare the data. Reverse transcription-quantitative polymerase chain reaction (RT-qPCR) and nanostrings provide information only about selected transcripts producing selection bias, compared to less selective microarrays and high-throughput RNA sequencing (RNAseq).

The difficulties summarized above precluded formal meta-analyses of transcriptomic data in MF, necessitating manual data synthesis based on the binary assessment of the expression of single genes (upregulated vs downregulated) [24,25]. This paper is a systematic review focusing specifically on transcriptomic changes in MF. We stratified the data according to the type of MF lesion (plaque or tumor) and the nature of the comparator used to determine the differentially expressed genes.

## 2. Methods

**Figure 1:**
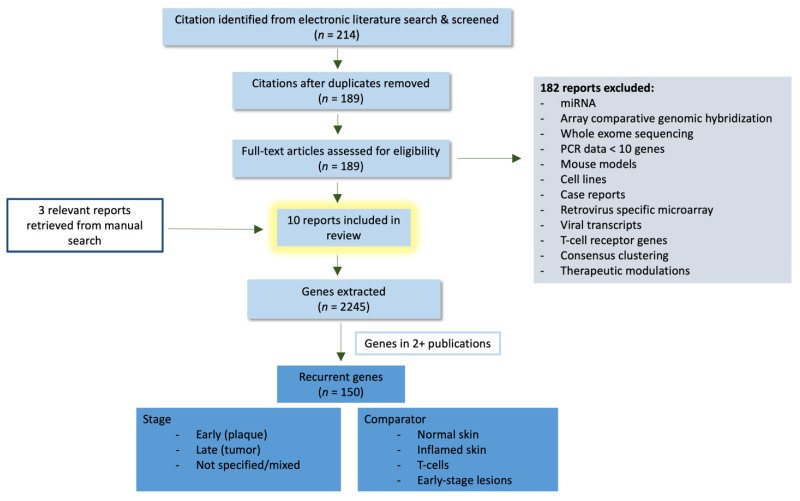
Workflow for obtaining pertinent genes. The paper selection was conducted via PubMed and the listed exclusion criteria were used to obtain relevant publications. The genes from the 10 publications were extracted and categorized in terms of stage and comparator.

**Figure 2:**
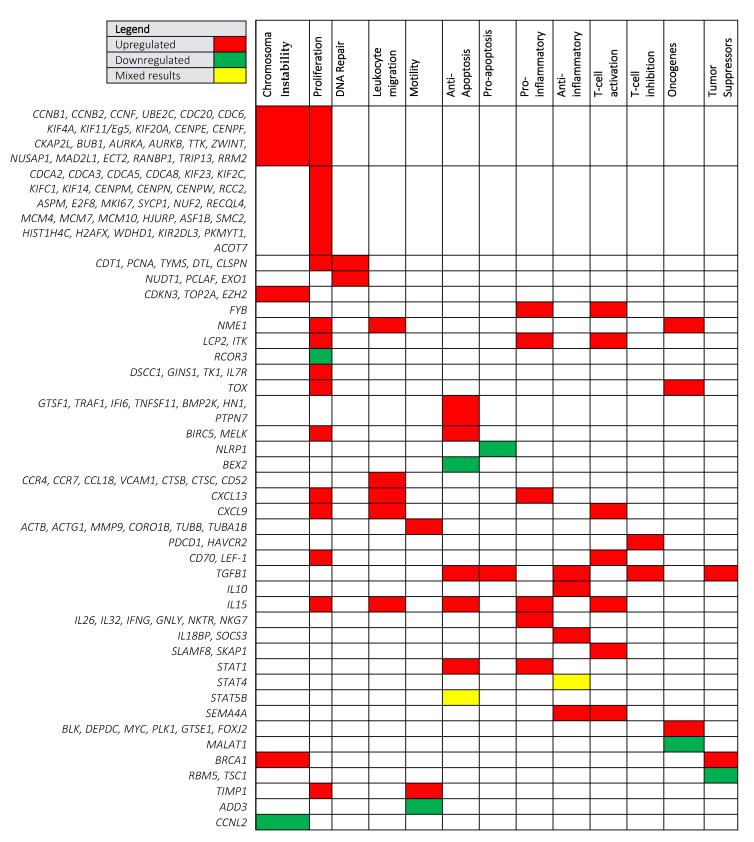
Differentially expressed genes in mycosis fungoides. The majority of the differentially expressed genes (*n* = 118) were categorized according to their function in Chromosomal Instability, Proliferation, DNA Repair, Leukocyte migration, Motility, Anti-Apoptosis, Pro-apoptosis, Pro-inflammatory, Anti-inflammatory, T-cell activation, T-cell inhibition, Oncogenes, and Tumor Suppressors. Although the remaining genes (*n* = 16) were differentially expressed, they could not be ascribed to an obvious function in MF: *NPHP3, ACVRL1, FUCA1, SECISBP2, CSAD, ATP5J2/ATP5MF, GOLGA8A, SLCO2B1, TESC, TTYH3, CLK4, cTAGE1, EIF5A, MRPL12, SNRPD1*, and *SCG2*.

We searched PubMed using the phrase “mycosis fungoides AND (transcriptomics OR microarray OR transcript*)” to identify potentially relevant citations. Once duplicates were removed, we had 189 publications; after examining for relevance using our predetermined exclusion criteria, 10 of 189 publications relating to MF transcripts were selected for data extraction (Figure 1). Studies that compared MF transcripts between arbitrary clusters of patients [26,27] were also excluded due to the variability in interpreting and comparing data from other sources. Furthermore, studies examining SS, which includes the dermatological manifestation seen in MF but also entails leukemic involvement, were excluded [28]. From the selected publications meeting our inclusion criteria, we manually extracted 2245 expressed genes (Supplementary Table S1), which were further refined to include only those genes that were reported in two or more publications, yielding 150 recurrent genes. We also extracted the skin lesion from which the biopsy was obtained (plaque, tumor, or not specified) and comparator (normal skin, inflamed skin, peripheral blood T-cells, MF lesional skin) for the 150 recurrent genes (Supplementary Figure S1). Plaques were defined as stages IA-IB, whereas tumors included all samples ≥IIB. Although MF skin samples comprise most of our study, 4 out of the 10 publications included CTCL skin samples but did not specify whether these samples were explicitly derived from MF or SS [17,18,19,20]. Due to the limited number of studies available, we further include those studies in our review, designating them as non-specified (NS) samples. By including only genes which appeared in more than one publication, we did not have genes that exclusively originated from NS samples, allowing us to eliminate the bias attributed towards their inclusion. Although the precise breakdown of differentially expressed genes in different sources against their comparator is reported in Supplementary Figure S1, for ease of interpretation, the 150 recurrent genes were categorized according to their function, irrespective of the source and comparator (Figure 2). Out of our 10 selected publications, three utilized microarrays [14,29], three—RNAseq [15,17,23], three—RT-PCR [18,19,24], and one study used single-cell RNA-sequencing for its analysis [20] (Supplementary Table S1). The vast differences in the methods of identifying transcript changes in these studies could also be a contributing factor for the limited number of recurring genes identified. 

## 3. Results

To determine the genetic profile of MF, 150 recurrent genes from the 10 papers were categorized based on the controls used and the type of lesion (plaque, tumor or non-specified) (Supplementary Figure S1). The majority of the differentially expressed transcripts were upregulated (140 genes), and only a few were downregulated (10 genes). 

### 3.1. Shared Cancer Pathways Upregulated in MF 

Previous analyses of global expression patterns across different cancers revealed that approximately 40% of the protein-coding genes were expressed in all cancer types, the rest showing a more tumor type-restricted expression [6]. The genes and pathways shared between the tumors are related to DNA replication, and regulation of apoptosis and mitosis. To map the transcriptomic changes in MF to known cancer-related pathways, we categorized the differentially expressed genes according to their function (Figure 2). As expected, the majority of differentially expressed genes were involved in cell proliferation (65 upregulated genes and 1 downregulated gene), chromosome instability and DNA repair, cell survival, and apoptosis. For the most part, we observed that oncogenes (*TOX, BLK, DEPDC, MYC, PLK1,* and *GTSE1*) were upregulated and tumor suppressor genes (*RBM5, TSC1*) were downregulated. Other noteworthy categories included upregulation of genes involved in leukocyte migration and motility, and chromosomal instability. Furthermore, there was an upregulation of anti-apoptotic genes (*BIRC5, TRAF1, IFI6, TNFSF11, MELK, BMP2K, HN1, TGFB1, IL15,* and *STAT1*) and downregulation of a pro-apoptosis gene (*NPHP3*), highlighting a possible mechanism employed by MF cells to aid in their survival. 

#### 3.1.1. Chromosomal Instability and Proliferation

Aberrant expression of cyclins is one of the most common disturbances in malignant cells. As highlighted in Figure 2, MF is associated with an increase in *CCNB1, CCNB2*, and *CCNF* and a decrease in cyclin *CCBNL2*. Cyclin B1, encoded by *CCNB1*, is consistently overexpressed in many malignancies such as breast, esophageal, gastric cancer, and lung cancer [30,31,32,33]. Cyclin B1 promotes the transition from the G2 phase to mitosis and its overexpression results in the bypass of the G2 DNA damage checkpoint [34], as has been documented by us in MF cell lines [35,36,37]. Overexpression of cyclin B1 and cyclin B2 (encoded by *CCNB2*) can induce aneuploidy through Aurora kinases A activation [38] and by inhibiting separase, a protein that typically functions to separate chromatids, leading to failure of chromatid segregation [38]. 

Interestingly, *CCNL2*, which encodes Cyclin L2, the only cyclin decreased in MF, induces apoptosis by upregulating tumor suppressors such as p53 and downregulating pro-apoptosis mediators such as Bcl-2 [39]. Thus, the observed downregulation of *CCNL2* may serve as a growth advantage contributing to MF pathogenesis. 

Additionally, increased expression for genes (*CDKN3* and *UBE2C*) that negatively regulate cyclins were also observed (Figure 2). For example, *CDKN3*, an inhibitor of CDKs, was upregulated in MF. Furthermore, *UBE2C* encodes an enzyme required for the ubiquitination of mitotic B cyclins. Although the protein encoded by *UBE2C* will decrease cyclins, its overexpression leads to chromosome missegregation and tumor formation [40]. This implies that it is not simply an increase or decrease in cyclins that is important in genomic instability, but rather timely targeted proteolysis via the ubiquitin–proteasome pathway. 

A recurrent feature of malignant cells is chromosomal instability (CIN), which results in a dramatic increase in DNA replication errors (20% in cells with CIN compared to 1% in normal cells) [41] and the deletions or duplications of chromosome segments [42,43]. We found 26 differentially expressed genes implicated in CIN, which may indicate that MF exhibits CIN, explaining the numerous chromosomal insertions and deletions often observed in this disease [7,44,45,46]. Several possible mechanisms of CIN can be proposed, based on the patterns of differentially expressed genes. Ten upregulated transcripts are implicated in merotely (*AURKB, BRCA1, CENPE, CENPF, KIF4, MAD2, RANBP1, CKAP2L, TOP2A, TTK,* and *ZWINT*), the mitotic error when a single kinetochore binds microtubules oriented toward both spindle poles [47,48]. Increased expression of *AURKA*, the Aurora kinase involved in the regulation of the cell cycle progression [49], may cause CIN in a variety of cancers [50,51,52]. The spindle assembly checkpoint (SAC) is recognized as another driver for CIN since deregulated SAC can lead to precocious separation of sister chromatids and an increase in chromosome missegregation [47,53]. We observed altered expression levels of SAC related components in MF such as *AURKB* [47,53,54], *KIF20A* [55], *BUB1* [47], *CDC20* [47,56], *CENPE* [47], *MAD2L1* [47,57], and *TRIP13* [58,59,60]. Finally, overexpression of the genes implicated in the failure of cytokinesis such as *EG5* [47] and *ECT2* [61] may disturb the final phase of the mitotic cycle. 

#### 3.1.2. Leukocyte Migration and Motility

As illustrated in Figure 2, various genes (*CXCL9, CCL18, CCR7, CD52,* and *MMP-9*) involved in leukocyte migration and motility were upregulated in MF. Chemokines are a family of chemoattractant cytokines that enable efficient leukocyte transendothelial migration and are critical regulators of skin-selective T-cell homing and lymphoma dissemination. Depending on the stage of the disease, dysregulation and significant shifts in the expression of chemokine–chemokine receptor axis genes, such as *CXCL9*, *CCL18*, and *CCR7*, mediate the progression of MF from early, skin-limited infiltrates to an advanced, disseminated cancer with systemic manifestations [62,63]. It is important to note that, in addition to their roles in cell migration and chemotaxis, chemokines receptors and their ligands also facilitate resistance to immune-mediated killing by activating prosurvival and anti-apoptotic pathways.

In low-grade MF, benign cells within early MF skin lesions (e.g., keratinocytes and dermal fibroblasts) express high levels of chemokines such as *CXCL9*, which preferentially attracts CD8 T-cells and T-helper 1 (Th1) cells that have the capacity to kill autologous malignant T-cells and represent an anti-tumor response in early disease [14,64]. Late-stage MF, however, is characterized by declining expression of *CXCL9* with concomitant increasing expression of T-helper 2 (Th2) associated chemokines such as *CCL18*. Specifically, studies have revealed elevated *CCL18* mRNA levels in MF tumor lesions and serum samples, compared with controls from healthy individuals [65]. Higher serum levels of CCL18 are linked to increased disease severity and poorer prognosis, most likely through promoting a Th2-dominated inflammatory microenvironment [66]. In addition, immunofluorescence staining revealed that CCL18 is preferentially expressed by macrophages in the invasion margin of the tumor [65], suggesting that CCL18 may be one facet in a series of complex, coordinated changes that facilitate malignant dissemination in late-stage disease. 

Previous microarray analyses have reported overexpression of the lymph node homing receptor *CCR7* in the dermis of tumor-stage MF [67]. Immunohistochemical staining of MF skin lesions further found that CCR7 expression correlated with subcutaneous extension of lymphoma cells [68]. As CCR7 mediates the physiological tropism of T-cells and metastasis of cancer cells to regional lymph nodes, these findings suggest that high expression of CCR7 may mediate lymphoid dissemination and spread of MF cells in late-stage tumor disease. Accordingly, advancing disease is associated with a gradual loss of epidermotropism, as the cytokine milieu can no longer retain lymphoma cells in the skin. 

Beyond the rise of these well-known chemokine and chemokine receptors, studies have reported higher levels of the anti-adhesion molecule *CD52* in lesional skin [22] as well as upregulation of the extracellular matrix-degrading enzyme *MMP-9* (matrix metalloproteinase 9) at the RNA level in advanced MF [22,69,70]. MMP-9 expression and secretion are primarily driven by MF cells and stromal cell populations in close vicinity to the tumor infiltrates. They are thought to play a role in facilitating tumor cell invasion and metastasis [69,70]. Collectively, these findings emphasize the complex and dynamic chemotactic signals that shape the tumor microenvironment and help traffic malignant T-cells to the skin and secondary lymphoid organs at different disease stages. 

#### 3.1.3. Pro-Tumorigenic Cytokines

As illustrated in Figure 2, emerging evidence supports the increasing expression of a range of pro-inflammatory cytokines during the course of the disease, including interleukin-15 (*IL15*) *IL32*, *IL10,* and *SOCS3*. These cytokines have been reported to play a pivotal role in suppressing cell-mediated anti-tumor responses while promoting a chronic pro-tumorigenic inflammatory microenvironment that fuels malignant T-cell proliferation. The cytokine IL-10 is elevated in malignant clones and is known to impair cellular anti-tumor response by virtue of its ability to inhibit the production of Th1 cytokine [71]. Studies have also found that IL-10 is associated with advanced disease, with lesional skin from the tumor stage showing higher expression of *IL10* mRNA, compared with patch and plaque stage MF [72]. Similarly, quantitative analysis of cytokines in MF lesions have given evidence for a stage-dependent increase of the pro-inflammatory cytokine *IL-15* and *IL-32* with disease progression [73,74]. It is hypothesized that IL-15 promotes tumor progression via induction of survival and anti-apoptotic signals in malignant T-cells [75], while IL-32 has been shown to accelerate the proliferation of CTCL cell lines through MAPK and NF-KB dependent mechanisms [76]. Finally, constitutive activation of the suppressor of cytokine signaling-3 (SOCS3) is thought to protect tumor cells against interferon alpha (IFNα)-mediated growth inhibition [77]. Taken together, MF progression is associated with shifts in a plethora of pro-tumorigenic cytokines, and further studies are required to gain a functional understanding of the tumor microenvironment and the treatment strategies that can restore immunosurveillance. 

### 3.2. Prognostic Cancer Pathways in MF

**Figure 3:**
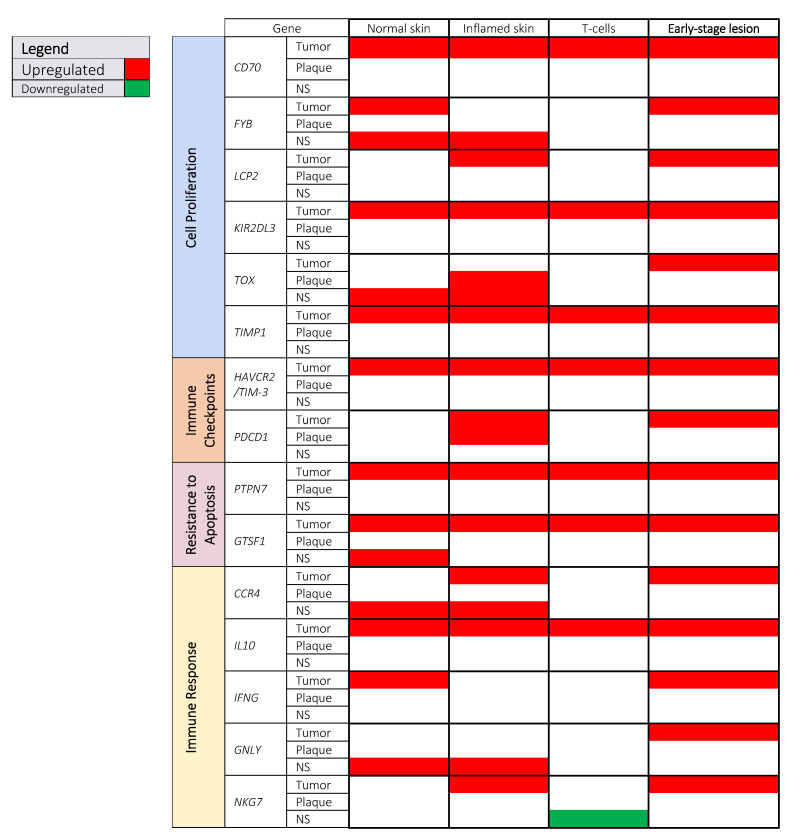
Genes involved in disease progression. A total of 15 genes were increased from early stage to late stage of MF which corresponded to genes involved in cell proliferation and activation, immune checkpoints, resistance to apoptosis, and immune response. Additional data from tumor, plaque, and NS samples against various controls (normal skin, inflamed skin, and T-cells) are represented to provide more detailed comparison regarding these 15 genes implicated in disease progression. Abbreviations: NS, non-specified/mixed.

There are no studies that link MF-specific mortality with genomic and transcriptomic alterations. However, the question of whether certain transcriptomic changes have prognostic importance can be answered indirectly by comparing the biopsies from early-stage disease (stage IA and IB) to the advanced stages (≥IB). We have extracted available data relevant to stage progression of MF and identified 15 genes which were categorized in the following way: cell proliferation (*CD70, FYB, LCP2, KIR2DL3, TOX,* and *TIMP-1*), immune checkpoints (*HAVCR2/TIM-3,* and *PDCD1*), resistance to apoptosis (*GTSF1,* and *PTPN7*), and immune response (*CCR4, IL-10, IFNG, GNYL,* and *NKG7*) (Figure 3). 

#### 3.2.1. Cell Proliferation

As indicated in Figure 3, MF disease progression was associated with the upregulation of genes involved in cell proliferation and activation (*CD70, FYB, LCP2, KIR2DL3, TOX,* and *TIMP-1*). Many of the genes differentially expressed between early and late-stage patients are relatively specific to the lymphocytes. CD70 is the TNF receptor ligand, which, upon its interaction with its receptor CD27, results in the activation of T-cell proliferation [78]. Expression of *CD70* in tumor cells from various hematological and solid malignancies is more commonly associated with poor prognoses [79]. Clinical trials utilizing the blockade of CD70 in CTCL have been carried out with a recent phase I trial showing that the treatment is safe, well-tolerated, and resulted in a 23% overall response rate, with some patients regressing from plaque to patch stage after treatment [80]. *FYB* and *LCP2* are T-cell adaptor genes involved in T-cell proliferation and activation [81,82]. Although their role in MF has not been investigated, they might be functionally significant, increasing the TCR-independent proliferation of tumor cells [16,17,19,26,83,84]. Overexpression of *KIR2DL3* has been described in Sezary syndrome and MF [85], but its functional role in MF is not fully elucidated. KIR2DL3 is likely to provide an activation signal via the Vav-1 and MAPK/ERK pathway, rendering the cells mitotically active [86,87]. 

*TOX* (thymocyte selection associated HMG-box) encodes a protein that is highly expressed in the thymus due to its involvement in aiding the transition of immature CD4+CD8+ double-positive T-cells to lineage-specific CD4+ single positive T-cells [88]. Upon leaving the thymus, Tox is downregulated in these cells, and thus peripheral T-cell subsets do not typically express this protein. Tox is ectopically expressed in CD4+ T-cells from MF lesions [89], with tumors expressing higher levels than patch stage lesions. Transfection of *TOX* in MF cell lines increased proliferation, migration, and invasion, further highlighting the oncological role of this gene [90]. Furthermore, increased immunohistochemical expression of TOX serves as a poor prognostic factor, as its expression is positively correlated with MF progression [89,91]. 

TIMP-1 is one of the tissue inhibitors of matrix metalloproteinases (MMP), the expression of which was previously linked with worse prognoses in melanoma [92], multiple myeloma [93], and breast cancer [94]. It is now recognized that TIMPs (tissue inhibitors of MMPs) have both MMP-dependent and MMP-independent mechanisms of action, with the latter acting as signaling molecules that alter gene expression that aid in oncogenesis [95]. Specifically, TIMP-1 can act as a growth factor in many types of cells, such as those seen in Hodgkin’s lymphoma [96,97]. Although an increase in *TIMP-1* gene expression is clearly observed in MF skin biopsies [17,20,23,24], the growth-promoting activity of the protein encoded by this gene is only speculation and, thus, more studies are required to ascertain the actual function of TIMP-1 in MF.

#### 3.2.2. Immune Checkpoints

MF is characterized by clonal expansion of malignant CD4+ T-cells that home to the epidermis [98]. This prompts the infiltration of tumor-infiltrating lymphocytes (TILs) and other peripheral blood mononuclear cells (PBMC) to the affected area of the skin to destroy the malignant CD4+ T-cells. However, it has been shown that CD8+ T-cells are unable to exert an effective cytotoxic action, resulting in immune tolerance against the malignant CD4+ T-cells [15]. Specifically, TILs in MF patients exhibit an exhausted phenotype, which is characterized by the upregulation of various co-inhibitory receptors such as PD-1 (program death 1, encoded by PDCD1) and TIM-3 (T cell immunoglobulin and mucin domain-containing protein 3, encoded by HAVCR2/TIM-3) [99]. Transient upregulation of co-inhibitory receptors on T-cells can serve as a homeostatic mechanism to maintain self-tolerance and prevent excessive immune response [100]. However, cancers create an environment whereby there is persistent antigenic stimulation, resulting in a sustained upregulation of various inhibitory receptors; this results in an impaired T-cell effector function such as cytotoxicity, proliferation, and cytokine production [101].

*HAVCR2/TIM-3* and *PDCD1* were not only increased with disease progression, but also displayed consistent levels of elevation in MF lesions (plaque and/or tumor) when compared to different controls (Figure 3). As most of the data obtained were from skin biopsies, which contain a mix of malignant and non-malignant cells, the cellular origin of those transcripts cannot be determined. However, one study showed an upregulation of *HAVCR2* and *PDCD1* from isolated CD8+ T-cells of MF lesions, which suggests that these co-inhibitory genes are predominantly associated with the non-malignant T-cells [15]. However, it is also known that MF cells frequently express PD-1 [99]. Specifically, the interaction of PD-1 with its ligand, PD-L1/L2, transduces a negative signal that inhibits T-cell proliferation and cytotoxicity, impeding effective anti-tumor response [102]. As a protective mechanism, tumors in MF have been shown to also express PD-L1 [103], with increased expression noted as the disease progresses [102]. Thus, an emerging strategy for the treatment of MF and other cancers has been to invigorate lymphocytes by developing antibodies against these inhibitory molecules. Nivolumab and pembrolizumab are two anti-PD1 therapies that have already been used for the treatment of MF. A phase I clinical trial with nivolumab showed an objective response rate (ORR) of 15% in patients with MF, but 0% in patients with other forms of CTCL, indicating the significant role that PD-1 plays in MF [104]. Similarly, a phase II clinical trial with Pembrolizumab found that MF patients had a significantly higher clinical response (ORR =57%) compared to SS (ORR = 27%) [105].

The co-inhibitory receptor TIM-3 has multiple ligands (e.g., Galectin-9 and phosphatidylserine), and its clinical significance in MF remains to be elucidated. However, clinical trials from other solid malignancies and lymphomas have indicated an enhanced response when TIM-3 blockade is used in conjunction with PD-1 inhibition [106]. Future research should focus on the role of Tim-3 in MF pathogenesis and the potential co-expression of PD-1 and TIM-3 in MF skin lesions.

#### 3.2.3. Resistance to Apoptosis

Next, two genes involved in resistance to apoptosis, *GTSF1* and *PTPN7,* were increased with MF progression (Figure 3). Ectopic activation of various cell- and tissue-specific genes is a frequent phenomenon in cancer because a global deregulation of epigenetic signaling causes unprogrammed gene activation [107]. Several meiosis-related cancer-testis antigens (meiCT) are aberrantly activated in MF. MeiCT antigens are normally present only in germ cells during oocyte development and spermatogenesis, and become transcriptionally silent in normal somatic tissues. The increase in expression of *GTSF1* and *STAG3* was reported in CTCL [16,17,19,108]. Ectopic expression of meiCT genes is suspected to play an essential role in maintaining cell survival through the inhibition of apoptosis (i.e., downregulating p53 and p21 tumor suppressor genes [24]) and promotion of chromosomal instability (i.e., generation of double-strand breaks leading to loss of heterozygosity and chromosomal arrangements) [108]. Another gene implicated in apoptosis resistance is *PTPN7*, as the knockout of this gene resulted in three times increase in apoptosis [109], while overexpression resulted in tumor morphological features in myeloid cells [110]. 

#### 3.2.4. Immune Response 

Lastly, the genes involved in immune response functions (*CCR4, IL10, IFNG, GNYL,* and *NKG7*) were also upregulated in late-stage MF (Figure 3). The chemokine receptor 4 (*CCR4*) is responsible for the trafficking of T-cells to the skin and MF cells have increased expression for this receptor, thereby allowing their accumulation in the skin [111]. High expression of CCR4 is a negative prognostic factor, as MF patients positive for CCR4 had a significantly lower survival rate than their negative counterparts [112]. Accordingly, CCR4 is a treatment target with the monoclonal antibody mogamulizumab [113,114]. Increased expression of IL10 has been suggested to play a role in the maintenance of the immunosuppressive skin environment within MF lesions and the polarization of MF cells towards the Th2 phenotype via signal transducer and activator of transcription 3 (STAT3) [115]. Constitutive activation of STAT3 can further promote Th2 phenotypes [116] and has been implicated in promoting the expression of co-inhibitory receptors such as PD-1 [117]. Surprisingly, the interferon γ (gene *IFNG*), a Th1 cytokine, was also upregulated in tumors and increased with progression. IFN-γ is known to have both beneficial and harmful effects in the tumor environment, depending on its concentration [118,119]. For example, excess IFN-γ signaling may lead to the apoptosis of tumor-specific CD8+ T-cells and induce genomic instability in tumors that leads to cancer progression [119,120,121]. Currently, the role of *IFNG* overexpression in MF is unclear.

Granulysin, the cytolytic protein encoded by *GNLY*, is present in cytotoxic granules of CTLs and NK cells, and is involved in inflammation, chemotaxis, and cytotoxicity [122]. Elevated expression of granulysin is associated with favourable outcomes in various cancers [123,124,125]; however, in MF, the granulysin is proposed to be an alarmin molecule attracting immature dendritic cells [126] which may promote immune tolerance to the tumor [127,128,129]. NKG7 is the pro-inflammatory protein aiding in lytic granule exocytosis from cytotoxic cells and is thus functionally synergistic with granulysin [130]. It should be noted that *NKG7* expression was downregulated in one of the studies (Figure 3), but this particular study analyzed plaque and tumor stages together, making the interpretation of their results unclear (Supplementary Table S1). Additionally, this was the only study within our cohort that utilized PBMC derived CD4+ T-cells in MF patients for the analysis, which is different from the rest of the studies that utilized skin biopsies.

## 4. Conclusions

This review was designed to be a thorough, systematic analysis of the gene expression profiles in the early and late stages of MF. Currently available data have severe limitations such as lack of standardization of the sample and the controls, as well as deficiency in reporting the clinical characteristics of the patient (stage, precise diagnosis, type of lesion). Therefore, only descriptive data presentation was possible. Additionally, several of the studies included in our systematic review discussed treatments that patients had undergone around the time of sample collection, which serves as a significant confounding factor (Supplementary Table S1). Some of these treatments included romidepsin and vorinostat, which are histone deacetylase (HDAC) inhibitors, enzymes that lead to alterations in chromatin structure and play a key role in epigenetic regulation of gene expression [131]. Additionally, synthetic folate analogs such as pralatrexate have anti-tumor effects and play a role in tumor reduced folate carrier (RFC-1) gene expression [131]. These treatments, and various others listed in Supplementary Table S1, may influence gene expression and represent a limitation when assessing the transcriptomic data.

Out of 2245 differentially expressed genes in MF found in the literature, we reduced the expression data set to only 150 genes which were reported in at least two independent studies. Most of those genes were associated with the already characterized pathways activated across many types of cancer: DNA repair and chromosomal stability, cell cycle and proliferation control, apoptosis, cell survival, and immune response. Also, in agreement with previous analyses of cancer transcriptomes, the majority of genes were transcriptionally activated, and only a few differentially expressed genes showed lower expression than the controls.

The more interesting question from the clinical point of view is the functional importance of the differentially expressed genes with respect to prognosis and response to treatment. This question could not be answered directly because survival and stage progression data are rarely available. However, by comparing expression patterns in the early stages of the disease and advanced lesions, we could identify 15 genes which might have prognostic significance. This very limited set of genes is not unexpected because, across different cancers, only a few genes have prognostic significance (e.g., in malignant melanoma only 20 of 13,491 expressed genes are linked to prognosis). These potential prognostic genes seem to be more restricted to lymphoid tissue and, specifically, to T-lymphocyte function and development (Figure 3). This confirms previous observations that prognostic genes appear to be more specific to only a few cancer types, and the genes detected across a large set of cancer samples seem to have a lower predictive value [6].

Currently, only a few treatments exist that specifically target proteins encoded by some of the 15 genes proposed to participate in MF progression. Of these treatments, only CCR4 inhibitor mogamulizumab is approved [114]. PD-1 inhibitors might be efficacious, although trials showed mixed responses [105,132]. KIR2DL3 was detected as upregulated, and a closely related inhibitory KIR3DL2 antibody has recently been tried for CTCL in Phase 1 trial [133]. Further understanding of what genes may play a role as a prognostic variable in MF may lead to more targeted treatments that participate in disease progression.

Probably the largest deficiency which impedes the progress in biomarker development in MF is the lack of reproducible reporting of patient-level clinical data. Our systematic review has found a lack of consistency in the sampling, sample preparation methods, and evaluation of the results from the study. These differences, though significant, can still be accommodated to derive conclusive markers with sufficient sample numbers. The number of samples studied for transcriptomic evaluation is largely limited. Additionally, published studies do not include adequate background information such as stage, disease progression, patient characteristics, and survival information. This can be addressed by a rigorous peer review system that requires authors to provide sufficient patient information for other groups to corroborate similar findings. However, without the clinical information including the diagnosis, the stage of the disease, and the clinical description of the biopsied lesion, it is impossible to meaningfully correlate the genotype/transcriptome with the prognosis. Broad utilization of publicly accessible platforms such as TCGA or The Human Protein Atlas would accelerate the identification of prognostic genes relevant for MF. 

## Data Availability

Data sharing is not applicable to this article as no new data were created or analyzed. All peer reviewed studies were accessed via PubMed.

## Supplementary Materials

The following are available online at https://www.mdpi.com/article/10.3390/cells10061409/s1, Table S1: Raw data of 2245 expressed genes gathered from the 10 studied publications, Figure S1: The categorization of 150 recurrent genes selected from the 10 publications.
